# Lactic acid bacteria: a promising alternative for recombinant protein production

**DOI:** 10.1186/1475-2859-11-157

**Published:** 2012-12-12

**Authors:** Elena García-Fruitós

**Affiliations:** 1 Institut de Biotecnologia i de Biomedicina, Universitat Autònoma de Barcelona, Bellaterra (Cerdanyola del Vallès), Barcelona, Spain; 2 CIBER de Bioingeniería, Biomateriales y Nanomedicina (CIBER-BBN), Bellaterra (Cerdanyola del Vallès), Barcelona, Spain

## 

Even though the use of Lactic Acid Bacteria (LAB) is well documented for a variety of dairy food fermentation dating back to the earliest written records [1,2], the use of these Gram-positive anaerobic microorganisms as recombinant microbial cell factories has taken place during the last decades. In this context, it is important to note that the enormous potential of these Generally Recognized As Safe (GRAS) organisms by the US Food and Drug Administration combined with the development of biotechnological, genomic and proteomic tools experienced during last years are expected to convert these microorganisms in emerging platforms for a wide range of applications [2,3]. Nowadays it is widely accepted that LAB-derived products from the industrial manufacture of fermented food such as milk products, vegetables, meat and wine [4,5], as well as lactic acid, antimicrobial peptides and high-value metabolites, are by far the most important LAB applications from an economical point of view. Besides, the use of these organisms as probiotics has also experienced an important increase in the last decades [6-10]. However, it is also important to stress that LAB characteristics make these organisms an ideal bacterial expression system for both homologous and heterologous proteins, including membrane proteins [11]. Interestingly, apart from the cheap and easily scalable protein production associated to the microbial nature of LAB, these species are food-grade expression hosts, that, contrary to what occurs in Gram-negative bacteria, do not contain endotoxins in their membrane, which are pyrogenic in humans and other mammals [12-14]. In this context, although *E. coli* is still the first-choice microorganism for the production of heterologous proteins, this species presents several obstacles, including, as mentioned, the presence of endotoxins in its derived products, limiting its final application as cell factory for the production of recombinant proteins, particularly those with pharmaceutical interest [15-17]. Thus, considering the limitations imposed by the use of *E. coli*, in the last years an increasing number of scientists are considering Gram-positive bacteria as a much optimal and safer microbial alternative for recombinant protein production. In fact, it is already possible to find commercially available enzymes produced in Gram-positive microorganisms [13]. Actually, *Bacillus subtilis* is nowadays one of the most used Gram-positive bacteria for the production of industrial enzymes, most of them being prokaryotic proteins [18]. Interestingly, *B. subtilis* is not only a convenient cell factory for the generation of safe products, but it also has an excellent secretion system, contrary to what occurs in *E. coli*, simplifying downstream processing of the protein and becoming a really interesting alternative [13,19]. Besides, several studies are being performed concerning *B. subtilis* quality control system, an approach that will significantly improve the successful production of difficult-to-express proteins in Gram-positive microorganisms in a near future [12,20]. Thus, Gram-positive bacteria are clearly promising candidates for the production of membrane and complex proteins, one of the most important challenges in Biotechnology, since these proteins are among the most significant drug targets. However, it should be mentioned that most of the efforts using safe hosts for recombinant protein production have been focused to the generation of recombinant homologous proteins and essentially using *B. subtilis*, being now time to extend our activities on the vast group of LAB for the production of heterologous proteins, those with a higher impact in Pharma and Biotech industries. In this context, the increasing interest on LAB has prompted researchers to develop key tools for recombinant protein production, such as suitable expression vectors, optimal promoters, modified strains and improved induction and secretion systems [1,11,18,21,22]. Interestingly, although recombinant protein production in LAB is far from being an extensively explored field, being much of their possibilities still to come to the surface, there are already examples in the literature showing the great potential of this safe expression system.

On the one hand, *Lactococcus lactis* has been described as another promising LAB in terms of recombinant protein production [18,21]. In this regard, Glenting and coworkers have shown that *L. lactis* can be a useful allergen expression system not only able to overcome the problems associated to the natural source extraction (low batch-to-batch variation, allergen preparations with defined purity and composition, among others), but also due to the safety of the isolated products [23]. Thus, recombinant production of genetically engineered hypoallergens for immunotherapy purposes is just an example of the high capacity of this group of LAB. Other examples describe the successful production of biologically active murine interferon-gamma, a cytokine that is commonly found forming inclusion bodies when expressed in *E. coli*[24], and a staphylococcal nuclease with biotechnological interest [22], published by Bermúdez-Humarán and collaborators and Tremillon and coworkers, respectively. Other publications prove that *L. lactis* is not only a good candidate for the heterologous production of both cytoplasmic and secreted proteins, but also an ideal alternative as a live bacterial vector able to efficiently deliver antigenic or therapeutic proteins at the mucosal level [21,25]. A review recently published by Bahey-El-Din and coworkers summarizes the already tested applications of *L. lactis* as expression host to deliver proteins with biomedical interest [26]. Analyzing the more than 40 different examples published in this paper, it is possible to conclude that *L. lactis* has successfully been used when delivering bacterial, viral and protozoal antigens and also therapeutic proteins from different origins including murine, human, ovine, rat and bovine [26]. Thus, these promising results consolidate the potential of *L. lactis* as a new vaccine delivery platform. Moreover, these data are also a clear prove of the potential of this group of Gram-positive bacteria for the production of both homologous and heterologous proteins, opening an amazing range of possibilities in the recombinant protein production field. Besides, since LAB are also able to produce biomolecules other than proteins, these microorganisms, and more specifically *Lactobacillus* genera, has also efficiently been explored as candidates for the delivery of functional agents and food ingredients for the production, among others, of gluten free bread with improved properties [27].

In summary, although many of the applications of LAB as a host factory need to be further explored, the already proved safety profile and efficiency of this expression system draw a promising future of GRAS organisms as the expression system of choice that can revolutionize the field of recombinant protein production.
